# Factors influencing the accuracy of multimodal image fusion for oral and maxillofacial tumors: a retrospective study

**DOI:** 10.1186/s12903-022-02679-0

**Published:** 2022-12-30

**Authors:** Lei-Hao Hu, Wen-Bo Zhang, Yao Yu, Zhi-Peng Sun, Guang-Yan Yu, Xin Peng

**Affiliations:** 1 Department of Oral and Maxillofacial Surgery, Peking University School and Hospital of Stomatology & National Center of Stomatology & National Clinical Research Center for Oral Diseases & National Engineering Research Center of Oral Biomaterials and Digital Medical Devices, No.22, Zhongguancun South Avenue, Haidian District, Beijing, People’s Republic of China; 2 Department of Oral and Maxillofacial Radiology, Peking University School and Hospital of Stomatology & National Center of Stomatology & National Clinical Research Center for Oral Diseases & National Engineering Research Center of Oral Biomaterials and Digital Medical Devices, Haidian District, Beijing, People’s Republic of China

**Keywords:** Oral and maxillofacial tumors, Multimodal image fusion, Accuracy, Influencing factors

## Abstract

**Background:**

Ensuring high accuracy in multimodal image fusion for oral and maxillofacial tumors is crucial before further application. The aim of this study was to explore the factors influencing the accuracy of multimodal image fusion for oral and maxillofacial tumors.

**Methods:**

Pairs of single-modality images were obtained from oral and maxillofacial tumor patients, and were fused using a proprietary navigation system by using three algorithms (automatic fusion, manual fusion, and registration point-based fusion). Fusion accuracy was evaluated including two aspects—overall fusion accuracy and tumor volume fusion accuracy—and were indicated by mean deviation and fusion index, respectively. Image modality, fusion algorithm, and other characteristics of multimodal images that may have potential influence on fusion accuracy were recorded. Univariate and multivariate analysis were used to identify relevant affecting factors.

**Results:**

Ninety-three multimodal images were generated by fusing 31 pairs of single-modality images. The interaction effect of image modality and fusion algorithm (*P* = 0.02, *P* = 0.003) and thinner slice thickness (*P* = 0.006) were shown to significantly influence the overall fusion accuracy. The tumor volume (*P* < 0.001), tumor location (*P* = 0.007), and image modality (*P* = 0.01) were significant influencing factors for tumor volume fusion accuracy.

**Conclusions:**

To ensure high overall fusion accuracy, manual fusion was not preferred in CT/MRI image fusion, and neither was automatic fusion in image fusion containing PET modality. Using image sets with thinner slice thickness could increase overall fusion accuracy. CT/MRI fusion yielded higher tumor volume fusion accuracy than fusion containing PET modality. The tumor volume fusion accuracy should be taken into consideration during image fusion when the tumor volume is small and the tumor is located in the mandible.

## Background

Multimodal image fusion, which provides different modalities of images integrated into a common reference frame under specific algorithms and displayed in one pair of multimodal images, has been frequently used in the diagnosis, virtual surgical planning, radiotherapeutic planning, and follow-up for oral and maxillofacial tumors [1–8]. Single-modality image sets like computed tomography (CT), magnetic resonance imaging (MRI), and positron-emission tomography (PET) can be registered and fused into one multimodal image set, which could reveal considerably more information regarding tumor infiltration and the spatial relativity between tumor and surrounding tissues than single-modality images.

The quality assurance of multimodal image fusion is the foundation of its in-depth application, and the core issue of quality assurance is ensuring high fusion accuracy [9]. Despite the wide application of multimodal image fusion, only a few of studies have focused on the accuracy of multimodal image fusion for oral and maxillofacial tumors. Most studies have demonstrated a fusion accuracy of ≤ 2 mm [9–13]. A previous study proposed a revised method to evaluate fusion accuracy that included the overall fusion accuracy—represented by mean deviation (MD) of six pairs of landmark points—and the tumor volume fusion accuracy—represented by Fusion Index (FI) [14]. The MD value ranged from 1.926 to 2.788 mm for different fusion algorithms, which showed a similar result of overall fusion accuracy with former studies. The FI value ranged from 0.520 to 0.594, and it was a newly proposed indicator of fusion accuracy that revealed to what degree the volume of the tumor on different modalities of image overlapped.

To achieve a relatively high accuracy of diagnosis or treatment planning by using multimodal image fusion, the fusion accuracy needs to be assured. Some researchers reported that the accuracy of multimodal image fusion was influenced by certain factors such as the parameters of single-modality image (i.e., pixel pitch and slice thickness) and the patients’ status when being scanned (i.e., the patient position and organ movement) [15, 16]. A previous study reported that multimodal image fusion that contained PET images seemed less accurate than CT/MRI fusion, which implied that the modality may be an influencing factor for fusion accuracy [14]. To our knowledge, no study has yet evaluated the potential influencing factors of the accuracy of multimodal image fusion for oral and maxillofacial tumors.

Ensuring high accuracy in multimodal image fusion for oral and maxillofacial tumors is crucial before further application. Therefore, this study explored the factors that influence the accuracy of multimodal image fusion for oral and maxillofacial tumors.

## Methods

### Patients and single-modality images

This study is a retrospective study and enrolled patients with a diagnosis of an oral and maxillofacial tumor who were referred to our department from January 2019 to January 2020. The inclusion criteria were as follows: (1) patients in whom the tumor was located in a deep oral area (e.g., gingiva of the posterior teeth, soft palate) or deep maxillofacial area (e.g., maxillary sinus, skull base, infratemporal fossa), and infiltrated at least two anatomical regions; (2) patients who had undergone at least two modalities of radiologic examination preoperatively and for whom complete Digital Imaging and Communications in Medicine (DICOM) files of at least two imaging modalities among regular CT (or contrast-enhanced CT, ceCT), MRI (T2 weighted or contrast-enhanced fat-suppressed T1 weighted), and PET-CT including maxillofacial area were available. The exclusion criteria were: (1) patients for whom the time interval between different radiological scans were over 20 days, as this might have led to tissue deformation caused by tumor growth; (2) the parameters of the patients’ image scans were ambiguous or could not be acquired from the DICOM files [14]. There was an overlapping in the patients enrolled in this study with the previously published works of the same research team, and this study expanded the sample sizes on that basis [14].

The study was approved by the Biomedical Institutional Review Board of Peking University School of Stomatology (approval number: PKUSSIRB-202054021).

### Multimodal image fusion

**Fig. 1:**
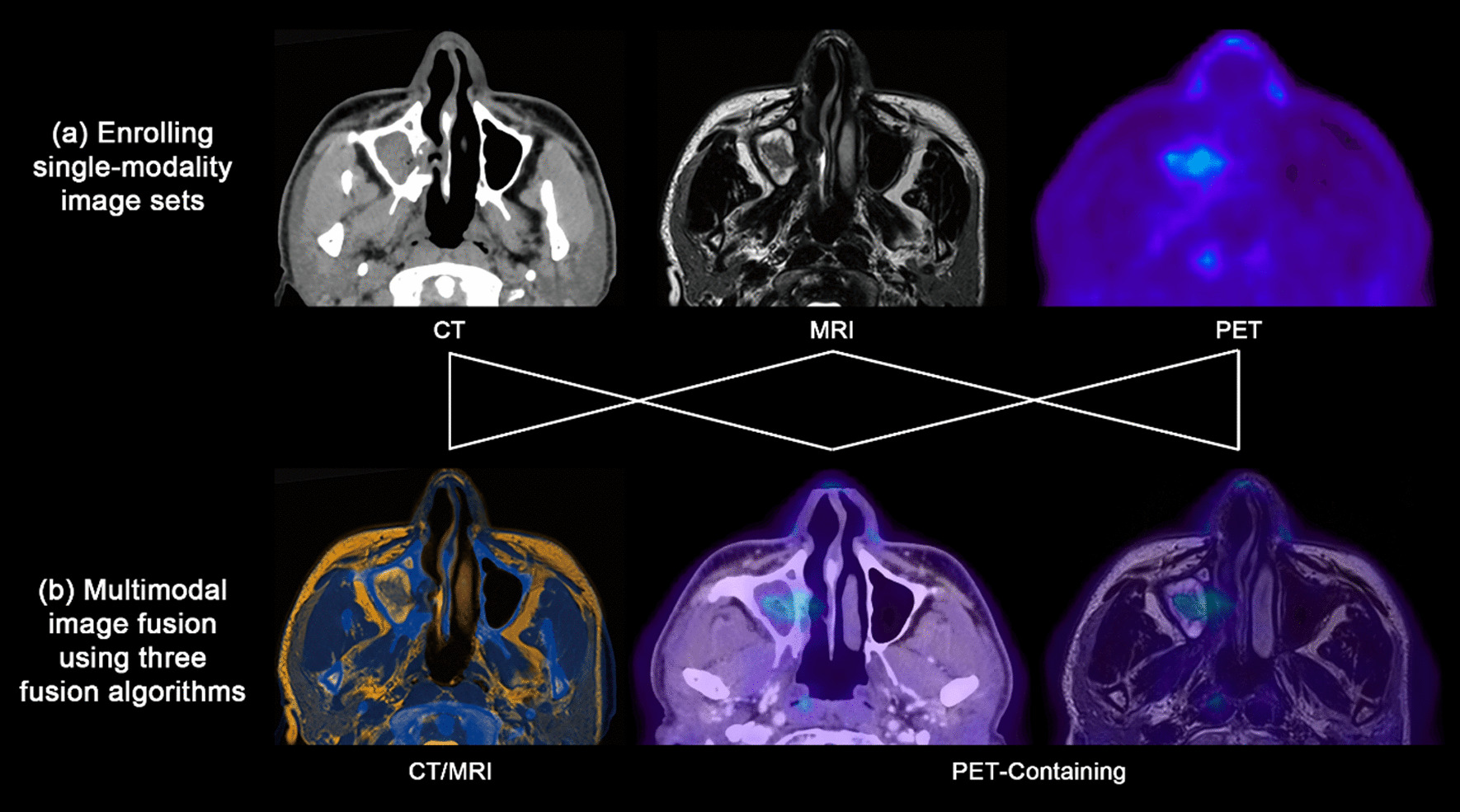
The schematic diagram of multimodal image fusion. **a** Three types of single-modality image sets were enrolled in this study. **b** The results of multimodal image fusion, which could be classified into CT/MRI image fusion and PET-containing image fusion according to the image modalities

The DICOM files of single-modality image sets of the same patient were imported into iPlan CMF 3.0 (BrainLAB, Feldkirchen, Germany) (Fig. 1a). By using “image fusion” module, two single-modality image sets were fused into one multimodal image set. The fusion modalities included two types: (1) CT/MRI image fusion (CT or ceCT image sets fused with MRI image sets); (2) PET-containing image fusion (PET-CT image sets fused with ceCT image sets, or PET-CT image sets fused with MRI image sets) (Fig. 1b). Three fusion algorithms were applied for every two single-modality image sets: (1) automatic fusion, finished automatically by the iPlan CMF software under the principle of maximization of mutual information in the region of interest; (2) manual fusion, finished manually by operating staff through translating or rotating one image set to align the other image set as much as possible; (3) registration point-based fusion: finished by iPlan CMF software through matching the corresponding registration points on two image sets that were marked by the operating staff before image fusion, and the registration points were all anatomical landmarks located at maxillofacial regions.

The process of image fusion was completed after two oral and maxillofacial surgeons (W.-B. Z., Y. Y.) with 8-year-experience in using the iPlan CMF software reached consensus.

### Evaluating the fusion accuracy

**Fig. 2:**
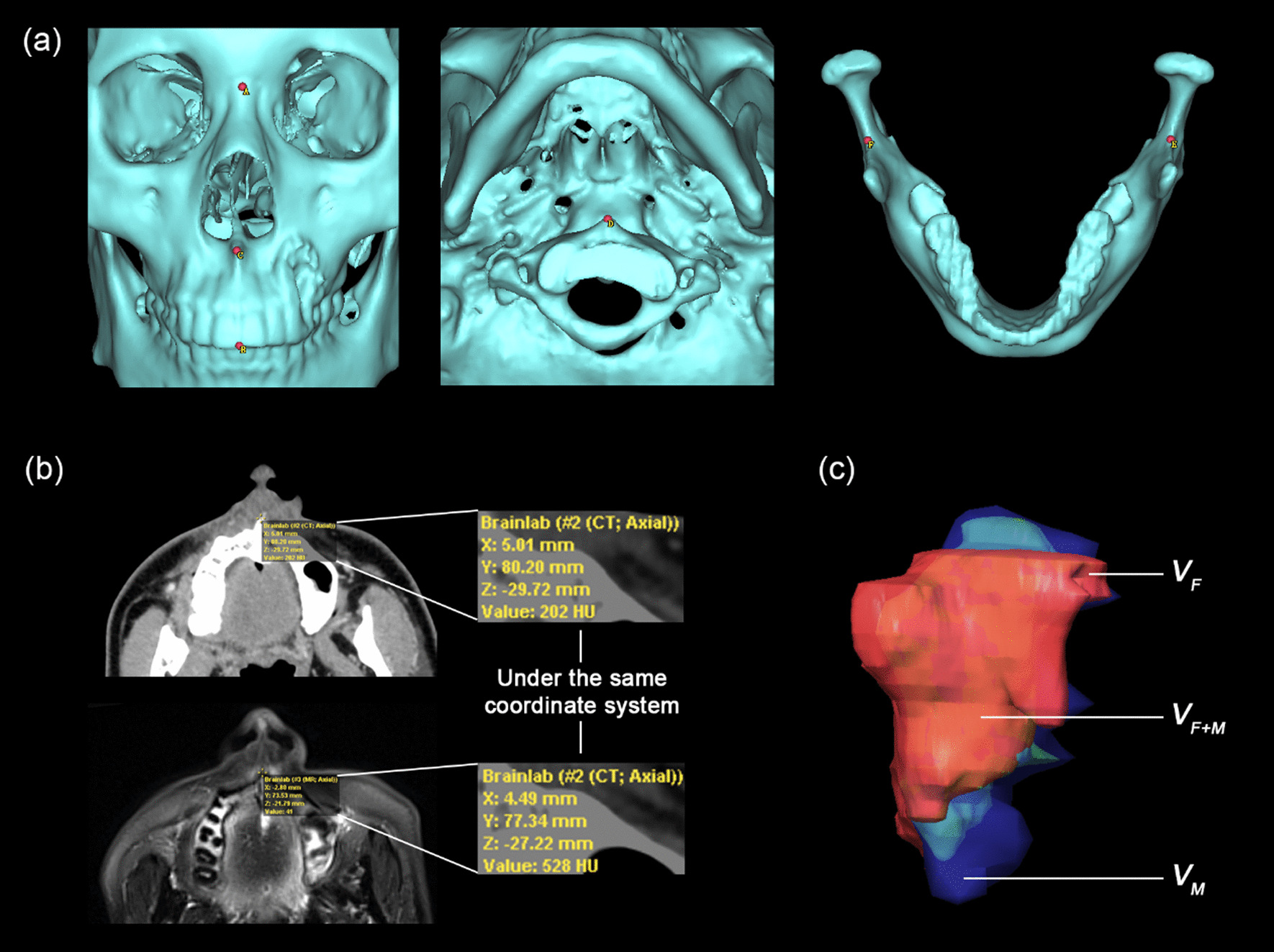
The schematic diagram of evaluating the fusion accuracy. **a** The location of the six pairs of anatomical landmarks. **b** The process of evaluating overall fusion accuracy which was represented as MD. **c** The process of evaluating the tumor volume fusion accuracy which was represented as FI

The accuracy of multimodal image fusion was evaluated for every fusion project, including overall fusion accuracy and tumor volume fusion accuracy (Fig. 2) [14].

To evaluate overall fusion accuracy, six pairs of anatomical landmarks were marked correspondingly on two image sets, three-dimensionally representing the superior, inferior, anterior, posterior, left, and right boundary of the overall image sets: (A) the interior point of nasion; (B) the tangency point of the upper central incisors; (C) the former point of the anterior nasal spine; (D) the former point of the atlas; (E) the tangency point of the left mandibular notch; (F) the tangency point of the right mandibular notch (Fig. 2a). The operating staff recorded the three-dimensional coordinates of six pairs of landmarks on two single-modality image sets under the same coordinate system. The coordinates of points on one image set were $\left( {x_{i1} ,y_{i1} ,z_{i1} } \right)$ ($i$ = 1, 2,…,6), while those on the other image set were $\left( {x_{i2} ,y_{i2} ,z_{i2} } \right)$ ($i$ = 1, 2,…,6) (Fig. 2b). The mean values of the coordinate differences of six pairs of landmarks along the x-, y-, and z-axes were $\Delta x$, $\Delta y$, and $\Delta z$, which were calculated as shown: $\Delta x = \left| {\frac{{\mathop \sum \nolimits_{i = 1}^{6} \left( {x_{i1} - x_{i2} } \right)}}{6}} \right|$, $\Delta y = \left| {\frac{{\mathop \sum \nolimits_{i = 1}^{6} \left( {y_{i1} - y_{i2} } \right)}}{6}} \right|$, $\Delta z = \left| {\frac{{\mathop \sum \nolimits_{i = 1}^{6} \left( {z_{i1} - z_{i2} } \right)}}{6}} \right|$. The overall fusion accuracy was represented by MD, which was calculated as shown: $MD = \sqrt {\Delta x^{2} + \Delta y^{2} + \Delta z^{2} }$. The MD value revealed the overall deviation of the same landmark points on two image sets. The lower the value of MD, the less the deviation between two image sets, and the better the overall fusion accuracy.

To evaluate tumor volume fusion accuracy, the tumor was delineated separately on two image sets, then the tumor volume on each image set ($V_{F}$ and $V_{M}$, presented in red color and in blue color) and intersected part of the tumor ($V_{F + M}$, presented in green color) were generated automatically (Fig. 2c). The tumor volume fusion accuracy was indicated by FI, which was calculated as shown: $FI = \frac{{V_{F + M} }}{{V_{F} }} \times \frac{{V_{F + M} }}{{V_{M} }}$. The higher the value of FI, the more the intersected part of tumor volume, and the better the tumor volume fusion accuracy.

The overall fusion accuracy and the tumor volume fusion accuracy were both evaluated twice by a well-experienced oral and maxillofacial surgeon (L.-H. H.) with 5-year-experience in using the iPlan CMF software and not participating in the image fusion process. The final result of fusion accuracy was the mean value of the two results. The evaluation process was finished under the guidance of a board-certified radiologist (Z.-P. S.) with 18-year-experience in radiological diagnosing of oral and maxillofacial tumors.

### Collection of characteristics and classification of variables

Patient and imaging characteristics were classified as follows: (1) Categorical variables including nature of the tumor, tumor location, dental artifact, change of patient’s position among different image scans, fusion modality, fusion algorithm and (2) Numerical variables including gross tumor volume, slice thickness, and pixel pitch of single-modality images. The value of gross tumor volume was acquired by averaging the tumor volume on different image sets that were generated automatically by evaluating tumor volume fusion accuracy.

### Statistical analysis

All measured data were analyzed using SPSS Statistics v24.0 (IBM Corp., Armonk, NY). MD and FI were set as the dependent variables. Univariate analysis was first carried out, including analysis of variance (ANOVA) for categorical variables and correlation analysis for numerical variables. Factors with *P* < 0.10 in the univariate analysis were included in the multivariate linear regression models to identify significant influencing factors. *P* < 0.05 in multivariate analysis was considered to indicate statistical significance.

## Results

### Overview of multimodal image fusion

Ninety-three multimodal image sets were generated by fusing 31 pairs of single-modality image sets. The basic characteristics of these 93 multimodal image sets are shown in Table 1.

**Table 1:** Basic characteristics of 93 multimodal images

Categories	Amount/Mean ± SD
Modalities	
CT/MRI	48
PET-containing	45
Fusion algorithm	
Automatic fusion	31
Manual fusion	31
Registration point-based fusion	31
Nature of tumor	
Benign	30
Malignant	63
Location of tumor	
Maxilla	57
Mandible	36
Dental artifacts	
Absent	78
Present	15
Change of patients’ position	
Absent	60
Present	33
Gross tumor volume (cm^3^)	31.67 ± 31.62
Interval days between different scans	7.76 ± 6.36
Thinner slice thickness (mm)	1.57 ± 0.70
Thicker slice thickness (mm)	3.03 ± 1.18
Lower pixel pitch (mm)	0.44 ± 0.13
Higher pixel pitch (mm)	1.92 ± 1.62

*SD* Standard deviation

### Univariate analysis

The results of univariate analysis are shown in Tables 2 and 3. Change of patient’s position among different image scans (*P* = 0.05) and thinner slice thickness (*P* = 0.01) were shown to potentially influence the overall fusion accuracy. Fusion modality (*P* < 0.001), nature and location of the tumor (both *P* < 0.001), and higher pixel pitch (*P* = 0.09) potentially influence the tumor volume fusion accuracy.

**Table 2:** Univariate analysis of potential influencing factors (categorical variables) of fusion accuracy

Characteristics	MD (mm)	FI
*Modalities*		
CT/MRI	2.06 ± 1.27	0.61 ± 0.14
PET-Containing	2.35 ± 2.17	0.41 ± 0.16
*P*-value	0.45	< 0.001*
*Fusion algorithms*		
Automatic fusion	2.63 ± 2.37	0.54 ± 0.17
Manual fusion	2.02 ± 1.33	0.48 ± 0.18
Registration point-based fusion	1.94 ± 1.35	0.50 ± 0.18
*P*-value	0.24	0.43
*Location of tumor*		
Maxilla	2.25 ± 1.33	0.63 ± 0.15
Mandible	2.17 ± 1.94	0.45 ± 0.16
*P*-value	0.81	< 0.001*
*Nature of tumor*		
Benign	2.05 ± 1.29	0.56 ± 0.16
Malignant	2.43 ± 2.32	0.42 ± 0.17
*P*-value	0.38	< 0.001*
*Dental artifacts*		
Absent	1.97 ± 1.55	0.55 ± 0.13
Present	2.24 ± 1.80	0.50 ± 0.19
*P*-value	0.59	0.37
*Change of patients’ position*		
Absent	1.89 ± 1.27	0.51 ± 0.14
Present	2.76 ± 2.32	0.50 ± 0.23
*P*-value	0.05*	0.79

*P < 0.10

**Table 3:** Univariate analysis of potential influencing factors (numerical variables) of fusion accuracy

	Characteristics	Gross tumor volume	Thinner slice thickness	Thicker slice thickness	Lower pixel pitch	Higher pixel pitch
MD	Pearson’s correlation coefficient	− 0.10	0.27	0.11	− 0.12	− 0.10
	*P*-value	0.33	0.01*	0.32	0.26	0.34
FI	Pearson’s correlation coefficient	0.36	− 0.01	− 0.07	0.18	− 0.15
	*P*-value	< 0.001*	0.90	0.50	0.09*	0.15

*P < 0.10

**Fig. 3:**
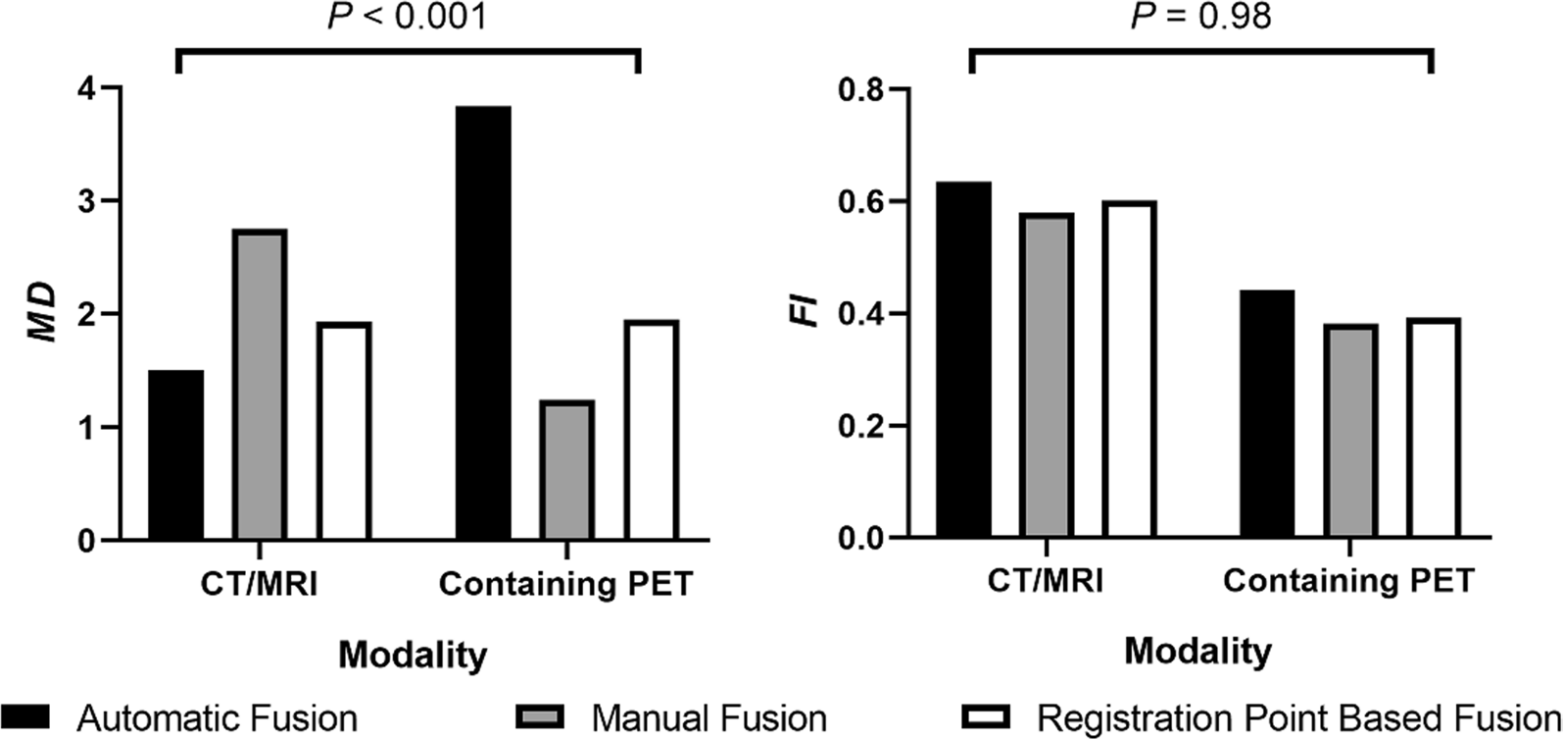
Fusion accuracy and two-way ANOVA of the interaction effect of modality and fusion algorithm

Per one-way ANOVA, neither the fusion modality nor the fusion algorithm was potential influencing factors of overall fusion accuracy, as their *P*-values were > 0.10. Nevertheless, the variation trend of the overall fusion accuracy of different fusion algorithms was opposite among different fusion modalities: for the CT/MRI modality, the overall fusion accuracy of automatic fusion was the best among three fusion algorithms, but it could be the worst when it comes to fusion modality containing PET. Such trend was not observed in tumor volume fusion accuracy (Fig. 3). This phenomenon implied that there was an interaction effect among fusion modalities and fusion algorithms, which could influence the overall fusion accuracy.

Therefore, two-way ANOVA was additionally performed, which verified the previous assumption: the interaction effect of modality and fusion algorithm was a potential influencing factor of overall fusion accuracy (*P* < 0.001) and would not influence the tumor volume fusion accuracy (*P* = 0.98) (Fig. 3).

### Multivariate analysis

#### Overall fusion accuracy

The model for multivariate linear regression analysis in which MD was set as the dependent variable was constructed using the variables that were significant in the univariate analysis (interaction effect of modality and fusion algorithm, change of patient’s position among different image scans, and thinner slice thickness) (Table 4). In the model, the *P*-value of the interaction effect of modality and fusion algorithm was < 0.05, which meant that the interaction effect among fusion modalities and fusion algorithms had significant influence on the overall fusion accuracy, manifested as:

1. Setting the overall fusion accuracy of automatic fusion in CT/MRI modality as the benchmark, both CT/MRI manual fusion (*P* = 0.02) and automatic fusion in the PET-containing modality (*P* = 0.003) could significantly decrease the overall fusion accuracy.
2. The overall fusion accuracy could be lower as the thinner slice thickness increased (*P* = 0.006).

**Table 4:** Multivariate analysis of potential influencing factors of overall fusion accuracy

Factors	β (95% CI)	Standardized beta coefficient	*P*-value
The interaction effect of modality and fusion algorithm			
CT/MRI × AF			
CT/MRI × MF	1.25 (0.33–2.17)	0.34	0.02*
CT/MRI × RPBF	0.43 (− 0.49 to 1.34)	0.12	0.40
PET-containing × AF	1.78 (0.81–2.75)	0.44	0.003*
PET-containing × MF	− 0.30 (− 1.27 to 0.67)	− 0.07	0.10
PET-containing × RPBF	0.29 (− 0.68 to 1.26)	0.07	0.70
Change of patient’s position	0.49 (− 0.18 to 1.15)	0.13	0.15
Thinner slice thickness	0.69 (0.21–1.17)	0.29	0.006^*^

*CI* Confidence interval, *AF* Automatic fusion, *MF* Manual fusion, *RPBF* Registration point-based fusion;

*P < 0.05

#### Tumor volume fusion accuracy

We set FI as the dependent variable of the model for multivariate linear regression analysis of tumor volume accuracy, while variables that were significant in the univariate analysis (modality, nature and location of tumor, gross tumor volume, and lower pixel pitch of single-modality images) were included as the independent variables (Table 5). In the model, the *P*-values of the modality, tumor location, and gross tumor volume were all < 0.05, showing that these factors could significantly influence the tumor volume fusion accuracy, manifested as:

1. The tumor volume fusion accuracy of the CT/MRI modality was better than modality containing PET (*P* = 0.01).
2. The tumor volume fusion accuracy was better in tumors located in the maxilla than in the mandible (*P* = 0.007).
3. The bigger the gross tumor volume, the higher the tumor volume fusion accuracy (*P* < 0.001).

**Table 5:** Multivariate analysis of potential influencing factors of tumor volume fusion accuracy

Factors	β (95% CI)	Standardized Beta coefficient	*P*-value
Modality			
CT/MRI			
PET-containing	− 0.12 (− 0.20 to − 0.03)	− 0.33	0.01*
Gross tumor volume	0.002 (0.002–0.003)	0.43	< 0.001*
Location of tumor			
Maxilla			
Mandible	− 0.10 (− 0.17 to − 0.03)	− 0.27	0.007*
Nature of tumor			
Benign			
Malignant	− 0.05 (− 0.13 to 0.03)	− 0.13	0.21
Lower pixel pitch	− 0.14 (− 0.35 to 0.07)	− 0.12	0.20

*CI* Confidence interval

*P < 0.05

## Discussion

This study explored the influencing factors of accuracy in multimodal image fusion for oral and maxillofacial tumors based on 93 multimodal images. The interaction effect of modality and fusion algorithm significantly influenced the overall fusion accuracy (*P* < 0.001). Manual fusion was not recommended in case of CT/MRI image fusion to obtain an accurately fused multimodal image, and automatic fusion was not recommended for PET-containing fusion.

When manual fusion was applied on CT/MRI image fusion, the operator needed to manually translate or rotate one pair of single-modality image set to align with another pair of single-modality image set in the region of interest. Automatic fusion and registration point-based fusion could decrease the errors resulting from determining the destination of translation or rotation manually by the operator in manual fusion, and therefore enhanced the overall fusion accuracy of multimodal image fusion.

According to the user’s manual of BrainLAB iPlan CMF, the principle of automatic fusion was maximization of mutual information. The software attained the fusion project with the largest mutual information as the final result of the automatic fusion [17, 18]. PET is a modality that reflects the metabolism of tissues and organs through the uptake of tracers in different parts of the body and displays tumor size, volume, and metabolic activity from the perspective of tumor metabolism. Unlike anatomical modalities such as CT or MRI, PET usually owns fewer gray levels, which makes it difficult to differentiate the anatomical structure outside the area of tumor volume. This can interfere with the calculation of mutual information of software platform, and decrease the mutual information, thereby resulting in a relatively low overall fusion accuracy [18, 19].

The tumor volume is a significant influencing factor of tumor volume fusion accuracy. When the operator carries out multimodal image fusion for small tumors, the tumor volume fusion accuracy should be paid more attention to. If the tumor volume fusion accuracy is not satisfied, fine adjustment of the location of image sets could be made based on aligning the tumor volume to different single-modality image sets.

The results of this study showed that reducing slice thickness could significantly improve the overall fusion accuracy of multimodal image fusion. Besides, pixel pitch was not a significant factor influencing the accuracy of multimodal image fusion. Some studies conducted preliminary investigations on the effect of spatial resolution on the accuracy of multimodal image fusion, and their conclusions were not the same. Ng et al. [20] used phantoms to explore the accuracy of transrectal ultrasound and cone-beam CT (CBCT) multimodal image fusion, suggesting that the slice thickness was not a significant factor influencing the accuracy. Kanakavelu et al. [21] used phantom and patient image data to verify the accuracy of the automatic fusion of kilovolt CT and megavolt CBCT, revealing that higher accuracy could be achieved when the slice thickness was 1 mm. Yang et al. [22] believed that in order to improve the accuracy of CT/MRI multimodal image fusion, CT and MRI image sets should be obtained within thin and consistent slice thickness. Based on the results of this study and previous studies, we could conclude that in multimodal image fusion containing certain kinds of image modality, image sets with thin slice thickness might be conducive to improving the overall fusion accuracy of multimodal image fusion compared to those with thick slice thickness. Nevertheless, it’s not clear whether decreasing the slice thickness could improve the accuracy of multimodal image fusion for all kinds of image modality, and it needed to be explored in the future.

Besides, the tumor volume fusion accuracy of the tumor located in the mandible was lower than that of the tumor located in the maxilla. The mandible is a movable structure. Because of malocclusion or edentulous jaws, it could not be guaranteed in some patients whether the upper and lower teeth were in the median position when undergoing different imaging scans, which resulted in the change of spatial position of tumors located in the lower jaw among different modalities of image sets and therefore reduced the tumor volume fusion accuracy [23].

Nevertheless, the sample size of this study was relatively small, and the influencing factors were obtained based on statistical methods, while the question regarding the mechanism involved in the influence of these factors on fusion accuracy remains largely unanswered, and further studies are needed.

## Conclusions

This study explored the influencing factors of the accuracy of multimodal image fusion for oral and maxillofacial tumors. To assure high overall fusion accuracy, manual fusion was not preferred in CT/MRI image fusion, and neither was automatic fusion in PET-containing image fusion. Using image sets with thinner slice thickness could increase the overall fusion accuracy. CT/MRI fusion yielded higher tumor volume fusion accuracy than PET-containing fusion. The tumor volume fusion accuracy should be taken into consideration during image fusion when the tumor volume is small and the tumor is located in the mandible.

## Data Availability

All data and materials as well as software application or custom code are available from the corresponding author on reasonable request.

## Abbreviations

CT: Computed tomography
MRI: Magnetic resonance imaging
PET: Positron emission tomography
MD: Mean deviation
FI: Fusion index
DICOM: Digital Imaging and Communications in Medicine
ceCT: Contrast enhanced computed tomography
ANOVA: Analysis of variance
CBCT: Cone-beam computed tomography
AF: Automatic fusion
MF: Manual fusion
RPBF: Registration point-based fusion
